# Azole-resistant *Aspergillus fumigatus* in the environment: Identifying key reservoirs and hotspots of antifungal resistance

**DOI:** 10.1371/journal.ppat.1009711

**Published:** 2021-07-29

**Authors:** Caroline Burks, Alexandria Darby, Luisa Gómez Londoño, Michelle Momany, Marin T. Brewer

**Affiliations:** 1 Plant Pathology Department and Fungal Biology Group, University of Georgia, Athens, Georgia, United States of America; 2 Plant Biology Department and Fungal Biology Group, University of Georgia, Athens, Georgia, United States of America; Rutgers University, UNITED STATES

## Abstract

*Aspergillus fumigatus* is an opportunistic human pathogen that causes aspergillosis, a spectrum of environmentally acquired respiratory illnesses. It has a cosmopolitan distribution and exists in the environment as a saprotroph on decaying plant matter. Azoles, which target Cyp51A in the ergosterol synthesis pathway, are the primary class of drugs used to treat aspergillosis. Azoles are also used to combat plant pathogenic fungi. Recently, an increasing number of azole-naive patients have presented with pan-azole–resistant strains of *A*. *fumigatus*. The TR_34_/L98H and TR_46_/Y121F/T289A alleles in the *cyp51A* gene are the most common ones conferring pan-azole resistance. There is evidence that these mutations arose in agricultural settings; therefore, numerous studies have been conducted to identify azole resistance in environmental *A*. *fumigatus* and to determine where resistance is developing in the environment. Here, we summarize the global occurrence of azole-resistant *A*. *fumigatus* in the environment based on available literature. Additionally, we have created an interactive world map showing where resistant isolates have been detected and include information on the specific alleles identified, environmental settings, and azole fungicide use. Azole-resistant *A*. *fumigatus* has been found on every continent, except for Antarctica, with the highest number of reports from Europe. Developed environments, specifically hospitals and gardens, were the most common settings where azole-resistant *A*. *fumigatus* was detected, followed by soils sampled from agricultural settings. The TR_34_/L98H resistance allele was the most common in all regions except South America where the TR_46_/Y121F/T289A allele was the most common. A major consideration in interpreting this survey of the literature is sampling bias; regions and environments that have been extensively sampled are more likely to show greater azole resistance even though resistance could be more prevalent in areas that are under-sampled or not sampled at all. Increased surveillance to pinpoint reservoirs, as well as antifungal stewardship, is needed to preserve this class of antifungals for crop protection and human health.

## The problem: Environmental azole use leads to drug-resistant *Aspergillus fumigatus*

Aspergillosis is a wide spectrum of respiratory conditions caused by the inhalation of spores from *Aspergillus* species [1]. The vast majority of cases worldwide (90%) are caused by the thermotolerant fungus *Aspergillus fumigatus*, followed by *Aspergillus flavus* (10% of bronchopulmonary cases), which is mostly reported in Asia, Africa, and the Middle East [2,3]. These fungi can affect both immunocompetent and immunocompromised individuals. The presentation of symptoms depends on host immunocompetency and the spore load inhaled [2]. The classification of aspergillosis disease can range from allergic bronchopulmonary aspergillosis (ABPA), to chronic pulmonary aspergillosis (CPA), to invasive aspergillosis (IA), which is the most severe and highly lethal [2,4,5]. ABPA is the most severe form of aspergillosis that can affect an atopic patient or a patient with cystic fibrosis and affects nearly 5 million people annually [4,5]. CPA encompasses the chronic inflammatory forms of aspergillosis, including aspergilloma, which most commonly affects patients with tuberculosis or other cavitary chronic lung diseases [4]. CPA was estimated to affect 3 million people annually in 2017, but it is also documented that the disease is underrecognized so incidence cannot be accurately estimated [4,5]. IA is the most severe form of aspergillosis, which affects severely immunocompromised patients, those under immunosuppressive treatments, or those undergoing surgery or a transplant [2,4]. IA is estimated to affect 250,000 people worldwide, and the incidence is increasing [5]; one study found that IA hospitalizations rose 3% in the United States between 2000 and 2013 [6], and another found that from 2013 to 2017, 4 times as many patients were admitted for IA per year as in 1996 [7,8]. This is likely due to the rise in immunosuppressive treatments for hematologic malignancies, stem cell transplants, and solid organ transplants, leading to neutropenic patients [2,6]. There has also been a shift in incidences of IA from neutropenic to non-neutropenic patients, such as those admitted for severe viral infections like influenza, and, more recently, Coronavirus Disease 2019 (COVID-19) [4,9]. In cases of IA that do not respond to treatment, mortality rates are between 50% and 100% [10]. Aspergillosis diseases are primarily treated using azole antifungals [11].

Azoles are a broad-spectrum class of antifungals that can be used on both human pathogenic fungi and plant pathogenic fungi [12]. Human-use azoles such as voriconazole, isavuconazole, and posaconazole are used to treat invasive forms of aspergillosis and other severe fungal infections [11]. Other azole drugs such as climbazole and clotrimazole are used to treat topical fungal infections [13]. Azole fungicides like tebuconazole and propiconazole are used against a variety of plant pathogenic fungi causing diseases such as powdery mildew, downy mildew, rusts, leaf spots, and flower blights [14]. Agricultural-use triazoles have become more popular in recent years. Tebuconazole and propiconazole use in the US alone increased over 4-fold from 2000 to 2016 [15]. Azoles are widely used in agriculture because they are inexpensive, they have a broad-spectrum systemic mode of action, and they are able to resist changes to their molecular structure so they can persist in the environment for an extended period of time [16]. Contamination of soil, wastewater, and sewage sludge with azoles also happens frequently, likely due to expulsion of topical azoles from urine and the skin [13]. Surface water and agricultural lands can be contaminated with azole biocides through incomplete removal from wastewater and use of sludge as fertilizer [13]. The wide use and persistence of azoles in the environment can contribute to the development of azole resistance in nontarget organisms, such as *A*. *fumigatus*.

Azole-resistant *A*. *fumigatus* was first discovered in the clinic in 1997 when analyzing isolates collected in the 1980s [17]. Azole antifungal drugs work by disrupting the synthesis of ergosterol, an important membrane lipid analogous to cholesterol in humans [12]. The production of ergosterol in fungi is facilitated by Cyp51A, a protein that converts intermediate sterols into ergosterol [12]. Azole resistance that develops in the clinic is generally caused by nonsynonymous nucleotide substitutions in the gene *cyp51A*, leading to amino acid changes such as G54E, P216L, and F219I [18]. These point mutations arise in patients treated with azoles, where there is high selection for azole resistance and an abundance of asexual growth and reproduction occur [18–20]. However, pan-azole–resistant strains, which are resistant to multiple azole drugs, were detected in azole-naive patients with aspergillosis in 2007 [21]. The TR_34_/L98H genotype of *cyp51A* was discovered to underlie pan-azole resistance in these strains [21]. This allele consists of a tandem repeat of 34 bases combined with a nonsynonymous nucleotide substitution, which leads to both the overexpression of *cyp51A* and a lower binding affinity of azoles for Cyp51A, respectively [21,22]. This allele became the dominant resistance mechanism in the Netherlands in 2008 and was then found in different unrelated patients in other parts of the world, which suggested that these alleles likely originated in environmental settings [23,24]. Cross-resistance to medical and agricultural azoles developed due to their similar structures and activities, as well as similar selective pressures for azole resistance in human hosts and in the environment when abundant reproduction by the fungus is accompanied by azole exposure [25,26]. A study by Snelders and colleagues [27] suggested that resistant isolates from the environment were likely moving into human hosts when they found that *A*. *fumigatus* isolates with the TR_34_/L98H allele were found in clinical settings and flower beds outside of the hospital and then commercial seeds, leaves, and compost. The environmental isolates were genetically similar to the isolates found in patients [27]. Since this study, there have been a plethora of sampling efforts across the world to determine where azole-resistant *A*. *fumigatus* is present in the environment. More alleles connected to the pan-azole resistance phenotype involving tandem repeats in the promoter region and/or nonsynonymous substitutions in *cyp51A* have since been discovered in both the clinic and the environment, including TR_53_, TR_46_/Y121F/T289A, TR_46_^3, and TR_46_^4 [28,29]. However, it is important to note that, although associations between environmental and clinical resistance have been presented, it has not been shown conclusively that patients acquire azole-resistant *A*. *fumigatus* from the environment.

*A*. *fumigatus* is primarily a saprophyte found on decaying plant matter and is, thus, commonly found in agricultural environments and on plant-based agricultural products [30–35]. This fungus also plays a notable role in carbon and nitrogen recycling in soil and compost heaps [36]. However, *A*, *fumigatus* is most abundant in areas where decomposition produces increased temperatures, such as in compost piles, decomposing plant material, and municipal waste treatment plants [37–39]. Other studies have found *A*. *fumigatus* to be prevalent in the outdoor air, flower beds, commercial potting mixtures, manures, and mulches [27,37,40,41]. Our aim for this review is to identify reservoirs (habitats where the fungus normally lives, grows, and multiplies) [42] of azole-resistant *A*. *fumigatus* by synthesizing published data on the environmental setting and substrate, the geographic locations, and the types of *cyp51A* alleles, if known, associated with azole-resistant *A*. *fumigatus* found in the environment. Additionally, we present an interactive global map showing where resistance has been detected. This will aid in characterizing and tracking the environmental settings where resistance is most prevalent and in identifying environments with azole-resistant *A*. *fumigatus*. Overall, this information will be helpful in identifying factors leading to the evolution and prevalence of azole-resistant *A*. *fumigatus* in the environment.

### Summarizing and synthesizing published data on azole-resistant *A*. *fumigatus* in the environment

**Fig 1:**
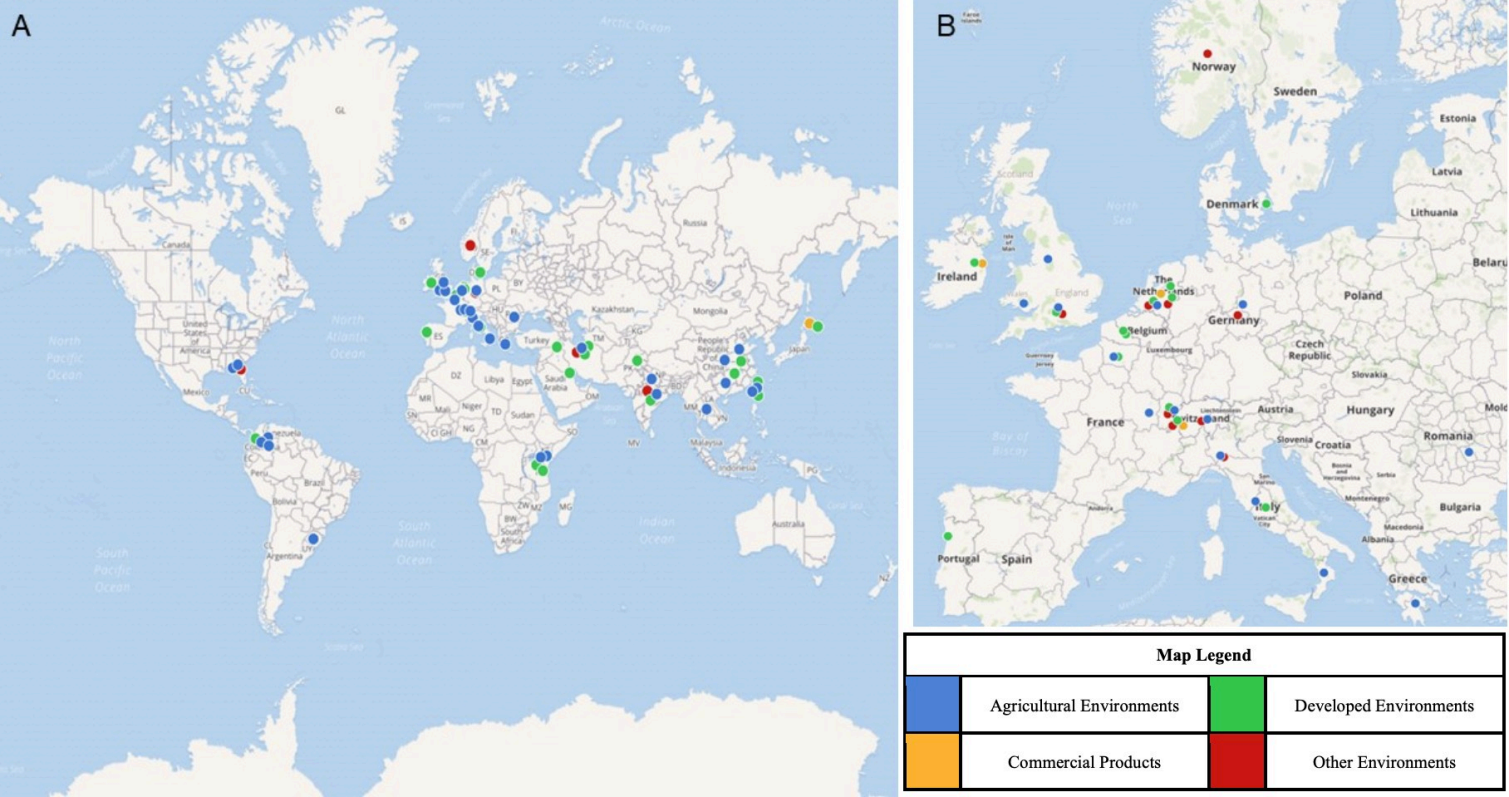
Azole-resistant *Aspergillus fumigatus* in the environment detected throughout **(A)** the world and **(B)** in Europe. The figure legend indicates different environmental settings from which resistant isolates were recovered. The digital map that includes additional environmental data, *cyp51A* alleles, and links to the published articles for each isolate can be found at https://maphub.net/cburks817/AfumMap. The base layer of the map can be found at https://maphub.net/map.

**Fig 2:**
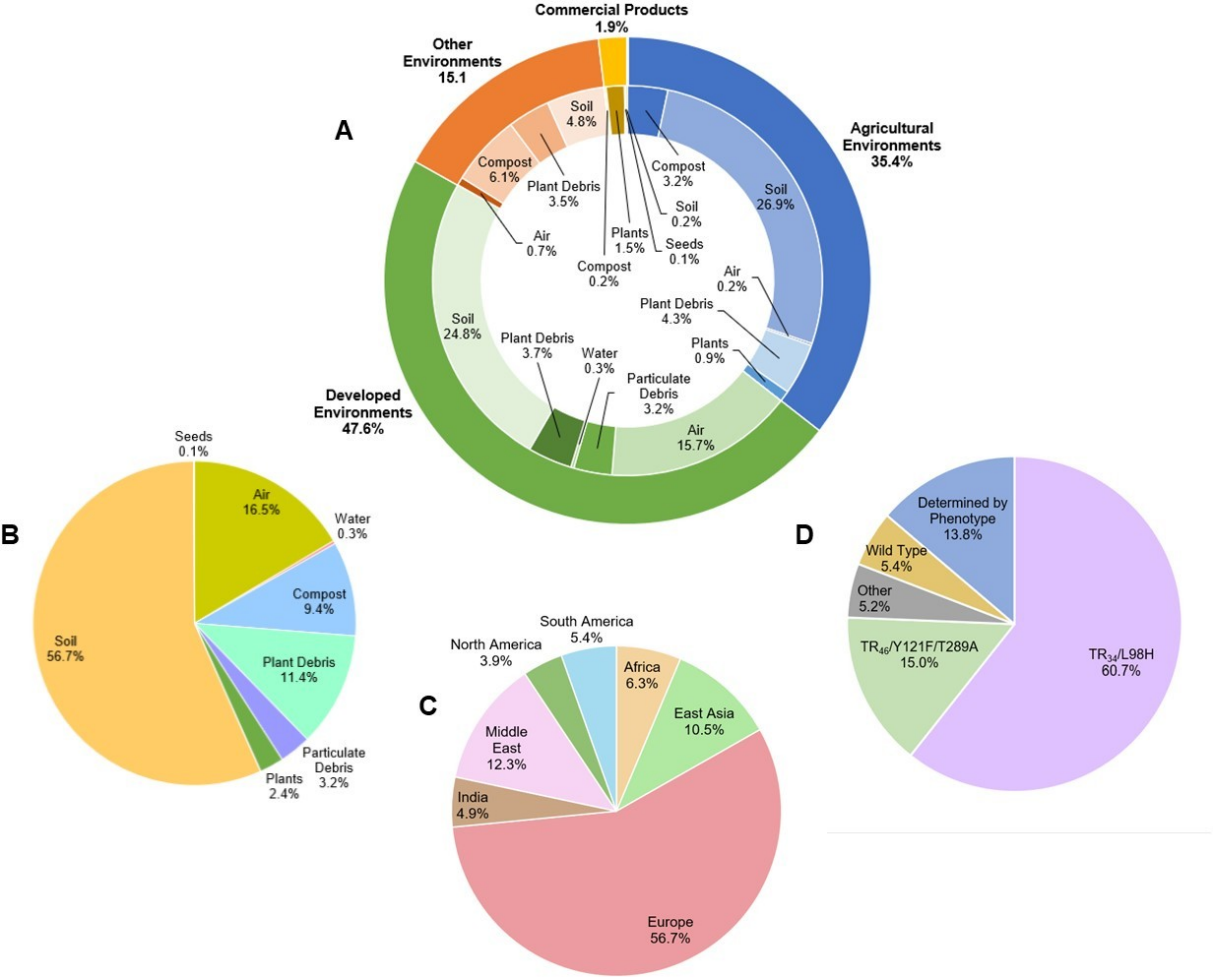
Frequency of azole-resistant *Aspergillus fumigatus* isolates detected in the environment by **(A)** environmental setting and substrate sampled, **(B)** substrate sampled across all environmental settings, **(C)** geographic region, and **(D)** *cyp51A* allele (“Other” includes less frequently detected *cyp51A* alleles and “Determined by Phenotype” includes resistant isolates that were not analyzed for *cyp51A* polymorphisms (S1 Table)).

Here, we summarize the published literature on azole-resistant isolates of *A*. *fumigatus* originating from the environment (S1 Table, Fig 1). Studies focused on isolates only from patients or that found no azole-resistant isolates were not included in our synthesis. To assist in summarizing and synthesizing the findings, resistant isolates were assigned to one of 4 possible environmental settings: agricultural environments, developed environments, commercial products, or other environments (S1 Table, Figs 1 and 2A). Isolates assigned to agricultural environments were collected from horticultural or crop production settings. Isolates assigned to developed environments were collected from public parks, gardens, homes, workplaces, or hospitals. Isolates assigned to commercial products were collected from retail products or products that would have been sold in a store. Isolates that could not be assigned to one of these 3 categories, for example, those from an isolated forest, were assigned to other environments. Resistant isolates were also assigned into categories based on the substrate sampled: air, compost, particulate debris, plant debris, plants, seeds, soil, and water (S1 Table, Fig 2B). Here, samples within the plant debris category include vegetable waste, woody debris, green waste, or other disposed plant parts. Particulate debris includes dust and swab samples from inside buildings. Resistant isolates were assigned to geographic locations of origin, including Africa, East Asia, Europe, India, the Middle East, North America, and South America (Fig 2C). Resistant isolates were separated by allele or phenotype, if *cyp51A* was not sequenced or no alleles that differed from wild type were present (Fig 2D). Isolates were assigned to the TR_34_/L98H or TR_46_/Y121F/T289A categories if these alleles were present in *cyp51A*. If the isolates had a different *cyp51A* allele from wild type that was neither of the 2 common alleles conferring pan-azole resistance, they were assigned to the other category. Finally, if isolates were found to have a wild-type *cyp51A* or *cyp51A* was not sequenced, they were placed into the determined by phenotype category. Available information for each azole-resistant isolate of *A*. *fumigatus* collected from the environment, including the published reference, environmental setting, substrate, the geographic origin, azole use in the environment, and *cyp51A* alleles, if known, was compiled to create an online interactive map (Fig 1, https://maphub.net/cburks817/AfumMap).

### Environments where azole-resistant *A*. *fumigatus* has been detected

Among the 52 published studies where azole-resistant *A*. *fumigatus* from the environment was detected (S1 Table), a total of 1,292 azole-resistant isolates were identified, and the specific environmental setting and substrate of 1,198 of the isolates were described. For the remaining 94 isolates, either the substrate was not mentioned [27] or the division of isolates between 2 different settings or substrates was not clear [24,43–45]. For the resistant isolates where the substrate was clearly described, the majority were detected in soil (56.7%), followed by air (16.5%), plant debris (11.4%), and compost (9.4%), with 3.2% in particulate debris, 2.4% in plants, 0.3% in water, and 0.1% in seeds (Fig 2B).

Most of the studies (31 of 52; 59.6%) focused on azole-resistant *A*. *fumigatus* in agricultural environments and 426 (35.6%) of the resistant isolates detected in the environment originated from agricultural samples (S1 Table, Fig 2A). Most resistant isolates from agricultural environments were recovered from soil (75.6%), followed by plant debris (12%), compost (9.4%), plants (2.6%), and air (0.5%). The crops grown in the agricultural settings sampled varied greatly; however, those that were sampled frequently included cereals, rice, maize, potato, strawberry, and flowers. A large portion of azole-resistant isolates from agricultural environments originated from fields of the crops listed above (50.2%), specifically flowers (27.7%). In total, 17 of the studies (54.8%) reported the use of azole fungicides in the agricultural settings sampled (S1 Table).

A total of 28 (53.9%) of the published studies found azole-resistant *A*. *fumigatus* in developed environments (some studies included isolates from more than 1 environmental category). Of the 570 (47.6%) azole-resistant isolates recovered from developed environments (S1 Table), most were recovered from soil (52.1%), followed by air (33.0%), plant debris (7.7%), particulate debris (6.7%), and water (0.5%) (Fig 2A). Resistant isolates from developed environments were collected and identified more from outdoor environments (53.9%) than indoor environments (46.1%). Of the isolates from developed environments, over half (58%) were identified from hospital environments, with 27.1% from the inside of hospital environments and 31.9% outside, but very near hospital environments. Moreover, 184 isolates from developed environments (32.6%) were found in gardens and flowerpots. Only 2 (7.1%) of these 28 studies reported the use of azole fungicides in developed environments [33,46]. These 2 studies found azole residues in tea gardens, hospital potted plants [46], green waste, and household waste [33].

A total of 4 (7.7%) of the studies where azole-resistant *A*. *fumigatus* was detected included retail or commercial products (S1 Table). From these 4 studies, 23 (1.9%) azole-resistant isolates were recovered (Fig 2A). Most of these isolates (78.3%) were recovered from live tulip and narcissus bulbs originating from the Netherlands [47,48]. The remaining azole-resistant isolates from retail products were recovered from commercial compost (8.7%) [27,48], commercial soil (8.7%) [49], and commercial seed (4.3%) [27]. Most resistant isolates detected in products were from flower bulbs [47,48], which have been identified as a hotspot of azole resistance [33,47,48,50,51].

The remaining 179 (14.9%) azole-resistant *A*. *fumigatus* isolates represented in 12 studies were sampled from other environments (S1 Table, Fig 2A). Furthermore, 5 of these studies performed sampling in locations that did not fit within the agricultural environments, developed environments, or commercial products categories: a forest, water treatment facility, soil filled with bird excrement, and 2 sawmills [46,52–54]. The other 7 studies sampled compost, soil, wood, and possibly other substrates but did not include details about the origin of their samples, preventing them from being assigned to an environmental category [24,27,33,49,55–57]. The studies examining wood and sawmills are especially intriguing because these areas are rarely studied as a potential for exposure to azole-resistant fungi despite the heavy treatment of wood products with azoles [33,52,53]. Most of the azole-resistant *A*. *fumigatus* isolates collected from other environments were recovered from compost (39.7%), followed by soil (32.4%), plant debris (23.5%), and air (4.5%) (S1 Table).

### Mechanisms of azole resistance in *A*. *fumigatus* from the environment

We were interested in determining which *cyp51A* alleles were most common among azole-resistant isolates from the environment. A total of 1,190 isolates (92.1%) were included in this portion of the data analysis (S1 Table). Resistant isolates were excluded if the specific allele was unclear for each isolate in the study or if the assignment of isolates across different environmental settings was unclear [24,43,58,59]. Most of the resistant isolates were found to have either the TR_34_/L98H allele (60.7%) or the TR_46_/Y121F/T289A allele (15.0%) (Fig 2D). The TR_34_/L98H allele was found on every continent with azole-resistant *A*. *fumigatus* and comprises a great proportion of the resistant isolates from environmental settings. Most of the isolates with TR_34_/L98H alleles were found in Europe (71.1%), followed by the Middle East (8.2%), India (7.8%), East Asia (5.1%), North America (4.2%), Africa (3.3%), and South America (0.4%). This allele was found in 29.3% of the azole-resistant environmental isolates in Africa, 37.4% of the resistant isolates in East Asia, 77% of the resistant isolates from Europe, 88.9% of the resistant isolates from India, 37.1% of the resistant isolates from the Middle East, 58.8% of the resistant isolates from North America, and 4.3% of the resistant isolates from South America.

The TR_46_/Y121F/T289A allele was also widespread and present in environmental azole-resistant *A*. *fumigatus* on every continent except Antarctica; however, it was far less common than the TR_34_/L98H allele. The TR_46_/Y121F/T289A alleles were especially prevalent in Europe (46.1%), South America (23.6%), and East Asia (14.0%) and were less prevalent in Africa (4.5%), India (3.4%), the Middle East (1.7%), and North America (6.7%). It was detected in 9.8% of the azole-resistant environmental isolates in Africa, 25.3% of the resistant isolates in East Asia, 12.3% of the resistant isolates from Europe, 9.5% of the resistant isolates from India, 1.9% of the resistant isolates from the Middle East, 23.5% of the resistant isolates from North America, and 60.0% of the resistant isolates from South America. This allele was especially common in South America, where the number of resistant isolates with this allele was far greater than the TR_34_/L98H allele [29,50,60].

The remaining azole-resistant isolates of *A*. *fumigatus* from the environment had *cyp51A* alleles other than the 2 common pan-azole resistance alleles or the *cyp51A* sequence was not characterized (Fig 2D). For these, we considered azole-resistant isolates based on minimum inhibitory concentration (MIC) data characterized by the European Committee on Antibiotic Susceptibility Testing (EUCAST) breakpoints: MIC values >1 μg/mL itraconazole and voriconazole, MIC values >2 μg/mL isavuconazole, and MIC values >0.25 μg/mL posaconazole [61]. Resistance to these antifungals often falls into areas of technical uncertainty, where the label of resistant depends on the patient being treated and other MIC values. In these cases, an isolate with MIC values = 2 μg/mL itraconazole, voriconazole, and isavuconazole and MIC values = 0.25 μg/mL posaconazole could be treated as susceptible [61]. However, for the purposes of this review, we used the 2020 strict EUCAST breakpoints to label isolates as resistant or sensitive.

A total of 64 of the isolates with azole-resistant phenotypes had wild-type *cyp51A* alleles (5.4%). Isolates with wild-type *cyp51A* alleles were found in Europe (43.8%), East Asia (37.5%), South America (15.6%), and the Middle East (3.1%). Resistant isolates with wild-type *cyp51A* alleles were not reported in the environments of Africa, India, and North America.

A total of 125 (10.5%) of the isolates had another *cyp51A* allele conferring resistance. The most common alleles other than TR_34_/L98H and TR_46_/Y121F/T289A were the TR_34_/L98H/S297T/F495I alleles (50; 4.2%) [27,33–35,53,62–64], G54E (22, 1.8%) [44,65], G448S (16; 1.3%) (both stand-alone and associated with the TR_34_, TR_46_, and TR_92_ alleles) [33,47,55,64], and M172V alleles (11; 0.9%) (both stand-alone and associated with F46Y) [34,64,66–68].

The majority of the TR_34_/L98H/S297T/F495I isolates originated from North America (30) [34], East Asia (16) [35,62–64], and Europe (4) [27,33,53]. The TR_34_/L98H/S297T/F495I alleles were found in compost [34], soil [35,63,64], plant debris [33,53], and air [27,62]. Most of the TR_34_/L98H/S297T/F495I isolates originated from agricultural environments [33–35,62,63], followed by developed environments [27,62,64] and other environments [53].

The G54E allele was most commonly found in Africa (13) [44], then Europe (8) [44,65], and India (1) [44]. The G54E allele was found in developed environments [44] followed by agricultural environments [44,65]. Isolates with the G54E allele were found in plant debris [44] and soil [44,65].

The M172V allele was most often found in Europe (5) [66–68], North America (5) [34], and East Asia (1) [64]. It was isolated from agricultural environments [34,68], followed by developed environments [64,66,67], then other environments [67]. It was most commonly found in compost [34], particulate debris [66], soil [64,68], and air [67].

The G448S allele was most common in East Asia (13) [47,64], Europe (2) [33], and the Middle East (1) [55]. The G448S allele was found in developed environments [64], commercial products [47], agricultural environments [33], and other environments [55]. Isolates containing the G448S allele were found in soil [64], followed by plants [47], plant debris [33], and compost [55]. Four studies also found uncommon tandem repeat alleles: 4 isolates with a TR_53_ allele and 2 with a TR_92_ allele [29,33,50,60].

Other tandem repeat polymorphisms were not commonly found in the environment. The TR_53_ allele was found in azole-resistant *A*. *fumigatus* in the environment in Bogota, Colombia, where it is associated with flower fields and agricultural fields [29,50,60]. The TR_92_ allele was found in flower bulb waste from agricultural fields in the Netherlands [33].

### Geographic distribution of azole-resistant *A*. *fumigatus* and azole use in the environment

Azole-resistant *A*. *fumigatus* has been found in the environment on every continent except Antarctica (S1 Table, Fig 1). Most of the azole-resistant environmental isolates were detected in Europe (56.7%), followed by the Middle East (12.3%), East Asia (10.4%), Africa (6.3%), South America (5.4%), India (4.9%), and North America (3.9%) (Fig 2C). The number of published studies covering each region was proportional to the number of resistant isolates recovered, except in East Asia, where 17.3% of the studies covered this region, but only 10.4% of isolates were found there. We examined the prevalence of azole-resistant *A*. *fumigatus* from different environmental settings within geographic regions; however, 3 studies were not included due to unclear assignment of isolates between settings or regions [24,44,62]. The majority of studies covering resistant isolates from Africa, East Asia, the Middle East, North America, and South America detected resistance in agricultural settings (S1 Table). Most studies focused on Europe and the Middle East detected resistance in developed environments. The majority of studies that detected resistance in commercial products and other settings were focused on resistant isolates from Europe.

To investigate if the geographic distribution and abundance of azole-resistant *A*. *fumigatus* in the environment is associated with environmental azole applications, we compiled available data on azole fungicide applications for each country [69–71] where resistant isolates have been detected (Table 1). Countries in Europe and East Asia applied more azole fungicides per hectare of agricultural land, whereas India, Africa, the Middle East, and South America had lower amounts of azole fungicides applied per hectare of land (Table 1). It is important to note that, except for 2018 data from Iran, the data from most of these countries with low usage reported are not recent, and it may not accurately reflect current azole fungicide application levels. Unfortunately, more recent data are not available. It is also important to note that azole fungicide use is not the only method where environmental *A*. *fumigatus* can be exposed to azoles; contamination of soil and surface water with azole biocides is a probable yet understudied method of exposure to azoles [13]. Data representing individual applications of azole fungicides would likely provide a more solid link between instances of azole resistance and fungicide use; however, that data are not easily collected and compiled. Therefore, we use countrywide data of fungicide applications to represent collective instances of azole applications to extrapolate the effect of azole use on resistance development.

**Table 1:** Azole fungicide application in agricultural environments.

Country	Region	Year	Total azole fungicides sold (kg)	Total agricultural land area (1,000 ha)	Kg azoles per 1,000 ha	Citations
the Netherlands	Europe	2018	291,957	1,822	160.24	[69,70]
Germany	Europe	2018	1,859,970	18,295	101.67	[69,70]
France	Europe	2018	2,817,233	28,660	98.3	[69,70]
Japan	East Asia	2018	285,763	4,420	64.65	[70,71]
Denmark	Europe	2018	165,200	2,632	62.77	[69,70]
Romania	Europe	2018	750,140	13,414	55.92	[69,70]
United Kingdom	Europe	2018	964,482	17,687	54.53	[69,70]
China	East Asia	2016	26,700,000	528,553	50.52	[35,70]
Thailand	East Asia	2014	751,149	22,110	33.97	[70,71]
Italy	Europe	2018	414,936	12,451	33.33	[69,70]
Switzerland	Europe	2018	40,262	1,537	26.2	[69,70]
Norway	Europe	2019	20,869	986	21.16	[69,70]
Portugal	Europe	2017	72,751	3,603	20.19	[69,70]
Ireland	Europe	2018	67,046	4,516	14.85	[69,70]
Greece	Europe	2018	46,865	6,110	7.67	[69,70]
US	North America	2016	2,270,229	405,265	5.6	[70,72]
Iran	the Middle East	2016	135,171	45,954	2.94	[70,71]
Colombia	South America	2000	77,110	44,859	1.72	[70,71]
Kenya	Africa	2001	6,350	26,839	0.24	[70,71]
India	India	1999	24,494	181,021	0.14	[70,71]
Iraq	the Middle East	2000	907	8,300	0.11	[70,71]
Tanzania	Africa	1995	907	33,050	0.03	[70,71]
Kuwait	the Middle East	1998	0	143	0	[70,71]
Taiwan	East Asia	2018	Unknown	791	NA	[70,71]
Brazil	South America	2018	Unknown	236,879	NA	[70,71]

Total azole fungicide sales (kg), agricultural land area, and azole fungicides per land area are given for countries where azole-resistant isolates of *Aspergillus fumigatus* were detected.

The Netherlands has by far the greatest amount of azole fungicide use per hectare of agricultural land, followed by Germany and France (Table 1), and the majority of the azole-resistant isolates were found in Europe (Figs 1 and 2C). Pan-azole–resistant *A*. *fumigatus* suspected to arise from the environment was first found in the Netherlands in 1997 [21], which is consistent with the agricultural origin theory of azole-resistant *A*. *fumigatus* resulting from increased use of azole fungicides [27,73]. Interestingly, Iran reported the third greatest number of azole-resistant *A*. *fumigatus* isolates after the Netherlands and France but had one of the lowest amounts of azole fungicides sprayed per hectare of agricultural land (Table 1) [55,74–76].

### Conclusions and future directions

Azole-resistant *A*. *fumigatus*, which is a serious threat to human health, has been found on 6 continents and in multiple different environments around the world (Figs 1 and 2). We compiled data on 1,292 azole-resistant *A*. *fumigatus* isolates reported from 52 independent studies (S1 Table). Most of the studies where azole-resistant *A*. *fumigatus* were detected were performed in Europe, where most resistant isolates were found. Over half of the azole-resistant isolates originated from soil samples. More than half of the resistant isolates came from developed environments, with the majority from sites including flower gardens and hospitals, and 35.6% of the isolates came from agricultural settings, with the majority found in sites including flowers, cereals, rice, maize, potatoes, and strawberries. This finding was unexpected since azole fungicide use is reported more often in agricultural settings, and agricultural sites were sampled more frequently (S1 Table). Reasons this could be the case include plants in developed settings being sprayed with azoles before transplanting or that there are more azoles present via deliberate azole fungicide use or unintentional contamination by azole fungicides or biocides. There was a high frequency of azole-resistant isolates found in hospital environments, which included plants, flower gardens, and samples from the air, water, and surfaces both indoors and outdoors. Similarly, the presence of azole-resistant *A*. *fumigatus* in hospital environmental samples is likely not due to the use of azole antifungals in the hospitals, but rather due to the introduction of foreign material such as commercial compost on the grounds [27,46], imported tulips on the grounds [77], and other landscaping and potted plants where azoles may have been applied during the growth process [27].

A recently published study found that an azole resistance hotspot most often develops in the presence of decaying plant waste and high concentrations of azole residues [33]. A review paper published the following year expanded the definition of a hotspot to include “environment(s) in which: (1) the physical, biotic and abiotic conditions facilitate the growth of the fungus and from which the fungus can spread; (2) this growth can take place for prolonged periods and the fungus can complete all the stages of its growth cycle; and (3) azoles are present, in different concentrations sufficient to select in populations, and combinations” [51]. Environments such as flower bulb waste, green waste, and wood chippings have already been defined as azole resistance hotspots [33,51]. Based on the high quantities of azole-resistant *A*. *fumigatus* in agricultural environments, flower gardens, and hospitals, we suggest that these locations be considered as possible azole resistance hotspots.

Additional research focused on azole resistance development and high frequencies of resistant *A*. *fumigatus* in flower production, flower waste, and flower bulbs in both agricultural and developed environments is needed to determine the factors contributing to resistance in these settings. Whether flowers are a common site for resistance due to frequent movement of resistant isolates on plant material, selection pressure from high azole use, high use of mulch or compost, or other factors needs further investigation.

Additional studies focusing on the usage of azole fungicides and the presence of azole residues in developed environments is needed, because the amounts used or quantities present are not often measured or reported [33,51]. The greatest number of resistant isolates in developed environments came from sampling outdoors. The most common outdoor developed setting for resistant isolates was garden soil. This could be due to high amounts of azoles used on plants in nurseries before they are sold [27,51]. Alternatively, azole-resistant isolates could be introduced to these environments, and, even in the absence of azole applications or residues, they might increase in abundance due to genetic drift or an unknown fitness advantage. Azole-resistant isolates have been previously detected in substrates with no detectable azole residues [33]. There were also high quantities of azole-resistant *A*. *fumigatus* found inside and on hospital grounds around the world. This finding is concerning because, while not common, incidences of nosocomial aspergillosis has been known to occur, and azole-resistant *A*. *fumigatus* poses an extreme health risk to immunocompromised individuals that would likely be present in higher numbers in a hospital setting [4,78]. Although unlikely, the presence of azole resistance in hospitals environments could also indicate that aspergillosis patients are spreading the resistant isolates into the hospital environment via emission of spores. However, this is unlikely since person-to-person transmission has only been reported once, suggesting that the airborne emissions of patients are insufficient to colonize outside environments [79].

The most common *cyp51A* allele among resistant isolates is the TR_34_/L98H, which comprised almost 75% of the resistant isolates. This allele was the first linked to pan-azole resistance and has been linked to the overuse of environmental azoles [27]. South America is the one area of the world where TR34/L98H was not the most frequent allele, and the TR46/Y121F/T289A allele was more frequently detected. This may be a result of different crops grown in that area, different azole antifungals used, introduction and proliferation of isolates with the TR46/Y121F/T289A allele due to random processes, or differences in sampling.

There is a large gap in knowledge concerning azole-resistant *A*. *fumigatus* in retail products, where only 4 studies have been conducted to date. There was only 1 study we found that reported azole resistance in an environment with low human impact (a forest). This prompts the need for more studies focusing on environments far from human impact because the spread of azole resistance may have spilled over into these environments as well. Several studies have found that isolates could be spread through plant material to different regions [47,48,77] and that isolates from different regions and environmental settings are related [27,35,43,44,46,59,60,62,74,80–83], supporting the idea that azole-resistant *A*. *fumigatus* might spread via contaminated plant material and dispersal. It is also likely that azole resistance had multiple independent origins since multiple different resistance mechanisms are present within different isolates. Overall, azole-resistant *A*. *fumigatus* is being found more frequently in more environments. Additional sampling of underrepresented environments around the world is needed so that the prevalence of azole-resistant *A*. *fumigatus* can be more accurately documented and so the risks of encountering these areas can be quantified. Our comprehensive survey of published studies on azole-resistant *A*. *fumigatus* in environments worldwide has made clear the overwhelming need to identify sources of resistance and areas of the environment where resistant isolates are most prevalent.

## Supporting information

S1 Table Environmental setting, substrate, geographic origin, and cyp51A genotype of azole-resistant *Aspergillus fumigatus* reported in the environment. (XLSX)
